# Extracellular vesicles from human-induced pluripotent stem cell-derived mesenchymal stromal cells (hiPSC-MSCs) protect against renal ischemia/reperfusion injury via delivering specificity protein (SP1) and transcriptional activating of sphingosine kinase 1 and inhibiting necroptosis

**DOI:** 10.1038/s41419-017-0041-4

**Published:** 2017-12-11

**Authors:** Xiaodong Yuan, Dawei Li, Xiaosong Chen, Conghui Han, Longmei Xu, Tao Huang, Zhen Dong, Ming Zhang

**Affiliations:** 1 Department of Transplantation and Urology, Ren Ji Hospital, School of Medicine, Shanghai Jiao Tong University, Shanghai, China; 2 Department of Hepatic Surgery, Ren Ji Hospital, School of Medicine, Shanghai Jiao Tong University, Shanghai, China; 3 Department of Urology, Xuzhou Central Hospital, Xuzhou Medical University School of Clinical Medicine, Xuzhou, China; 4 The Animal Facility of Ren Ji Hospital, School of Medicine, Shanghai Jiao Tong University, Shanghai, China; 5 Transplantation Center of the Affiliated Hospital of Qingdao University, Qingdao, China

## Abstract

Renal ischemia-reperfusion is a main cause of acute kidney injury (AKI), which is associated with high mortality. Here we show that extracellular vesicles (EVs) secreted from hiPSC-MSCs play a critical role in protection against renal I/R injury. hiPSC-MSCs-EVs can fuse with renal cells and deliver SP1 into target cells, subsequently active SK1 expression and increase S1P formation. Chromatin immunoprecipitation (ChIP) analyses and luciferase assay were used to confirm SP1 binds directly to the SK1 promoter region and promote promoter activity. Moreover, SP1 inhibition (MIT) or SK1 inhibition (SKI-II) completely abolished the renal protective effect of hiPSC-MSCs-EVs in rat I/R injury mode. However, pre-treatment of necroptosis inhibitor Nec-1 showed no difference with the administration of hiPSC-MSCs-EVs only. We then generated an SP1 knockout hiPSC-MSC cell line by CRISPR/Cas9 system and found that SP1 knockout failed to show the protective effect of hiPSC-MSCs-EVs unless restoring the level of SP1 by Ad-SP1 in vitro and in vivo. In conclusion, this study describes an anti-necroptosis effect of hiPSC-MSCs-EVs against renal I/R injury via delivering SP1 into target renal cells and intracellular activating the expression of SK1 and the generation of S1P. These findings suggest a novel mechanism for renal protection against I/R injury, and indicate a potential therapeutic approach for a variety of renal diseases and renal transplantation.

## Introduction

Renal ischemia followed by reperfusion (I/R), caused by circulatory shock of different etiologies, or by anesthesia, surgery, or transplantation, is a major cause of acute renal failure (ARF)^1,2^. In spite of supportive therapies, the mortality associated with AKI remains high^3,4^. Our limited understanding of the complex cell death mechanism in the process of AKI impedes the development of desirable therapeutics^5^. For a long time, apoptosis was recognized as the main form of cell death that is responsible for renal dysfunction in AKI^6^. Therefore, strategies targeting the apoptosis pathway have been widely explored for AKI treatment^7^. Despite the substantial therapeutic effect in animal models, the efficient anti-apoptosis intervention strategies are still absented in clinic. This could be partly ascribed to our limited understanding of the complex cell death mechanism in the process of AKI. Necroptosis is a recently identified novel form of cell death contributing to numerable diseases and tissue damages^8–11^. Increasing evidence has suggested that necroptosis has an important role in the pathogenesis of various types of AKI^12–19^. However, the signaling pathways and main regulators of necroptosis in the process of AKI remain unclear.

Recently, the mesenchymal stem cells (MSCs) derived from human-induced pluripotent stem cells (hiPSCs) have been used in pre-clinical studies and showed better performance compared to the adult MSCs in terms of cell proliferation, immunomodulation, cytokines profiles, production of microenvironment modulating EVs, and secretion of bioactive paracrine factors^20,21^. It has been shown that hiPSC-MSCs can prevent I/R damage in the kidney, liver, and heart^22–26^. However, the underlying mechanism of the protective effect of hiPSC-MSCs is still unclear.

Extracellular vesicles (EVs) are membrane-contained vesicles released in an evolutionally conserved manner by cells including MSCs. EV-mediated signals can be transmitted by all the different biomolecule categories such as proteins and nucleic acids (mRNA, miRNA, and other non-coding RNAs)^27^. Over the past few years, evidence has been shown that EVs are widely demonstrated to be implicated in cellular signaling during renal regenerative and pathological processes and participate in kidney development and normal physiology^28–32^. Although many EVs mechanisms are still poorly understood, in particular in the kidney, the discovery of their role could help to shed light on renal biological processes which are so far elusive. Recently, EVs secreted from MSCs or stem cells have been shown to play a critical role in protection against I/R injury in the liver, kidney, and heart^26,33–37^. Whether hiPSC-MSC-derived EVs are implicated in the healing properties of MSC-derived vesicles in AKI has not yet been investigated.

In this study, we investigated the renal protective effect of hiPSC-MSCs-derived extracellular vesicles (hiPSC-MSCs-EVs) on renal I/R injury, as well as the underlying mechanisms. We demonstrated that hiPSC-MSCs-EVs could reduce renal I/R injury via transcriptional activating of sphingosine kinase (SK) 1 and inhibiting necroptosis. Our study represents a potential mechanism for renal protection and has important implications for new therapeutic approaches to acute kidney diseases.

## Results

### Generation of hiPSC-MSCs and characterization of hiPSC-MSCs secreted EVs

**Fig. 1:**
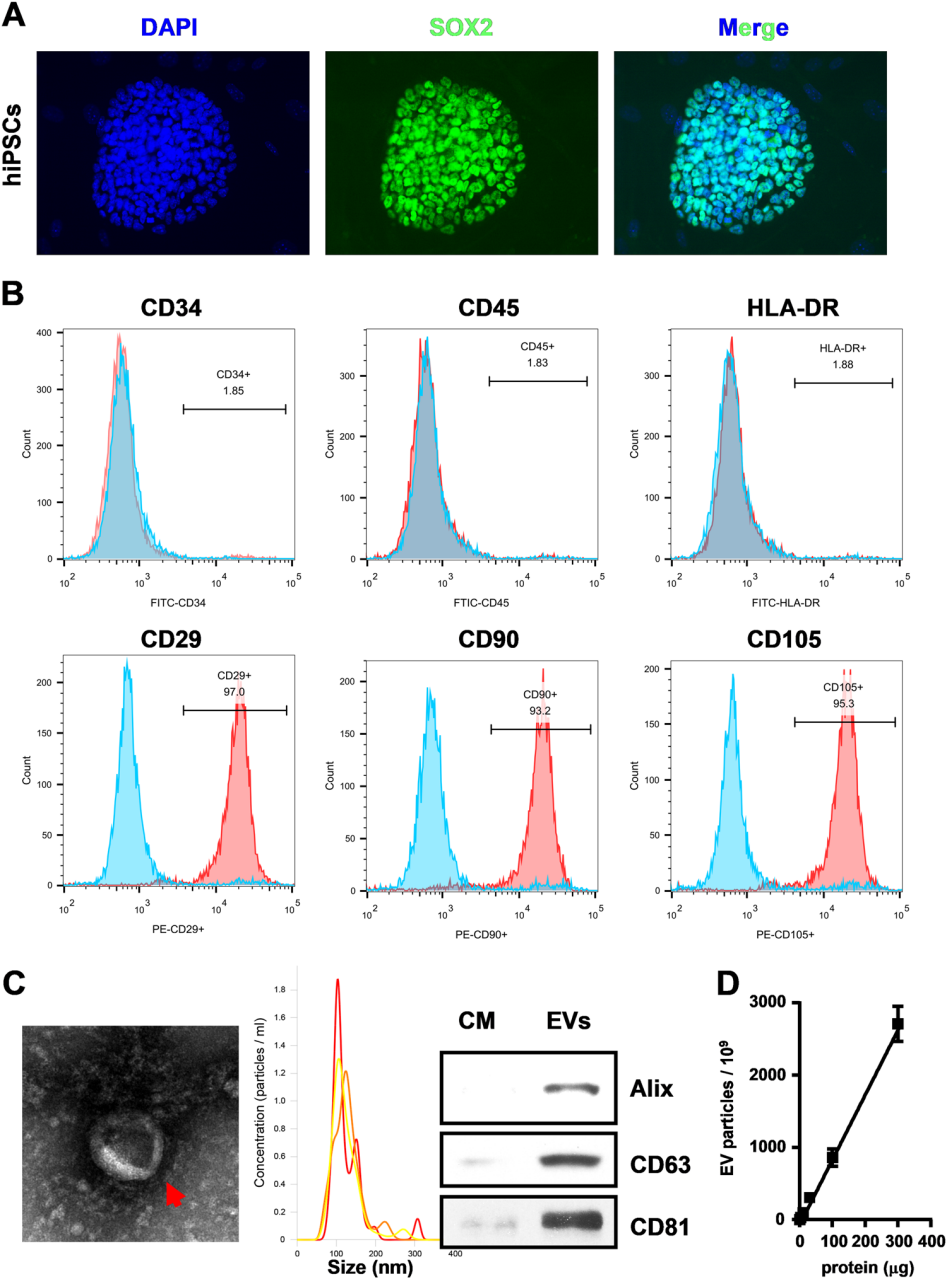
Characterization of hiPSC-MSCs and hiPSC-MSCs-derived EVs **a** Morphology of hiPSCs before MSC induction was examined by immunofluorescence staining. **b** Flow cytometric analysis of surface markers of hiPSC-MSCs. **c** Left: TEM images of EVs derived from hiPSC-MSCs (scale bar, 100 nm); middle: the sizes of hiPSC-MSCs-derived EVs (mean: 135 nm); right: western blot for Alix, CD80, and CD63 in hiPSC-MSCs-derived EVs. Medium alone is the control; **d** a standard curve indicated the relation between the protein content and the EVs number

Firstly, hiPSCs were successfully induced into hiPSCs-MSCs and grew in a monolayer with large spindle-shaped morphology at the colony border (Fig. 1a). Immunofluorescence staining was used to assess the surface antigens of hiPSCs (SOX2) before induction (Fig. 1a). Flow cytometry was also used to identify the surface antigens in differentiated hiPSCs-MSCs. The results showed that hiPSC-MSCs were negative for CD34, CD45, and HLADR, but positive for CD29, CD90, and CD105 (Fig. 1b). Furthermore, the EVs secreted from hiPSCs-MSCs were isolated and subjected to biochemical and biophysical analyses. Electron microscopy analysis on EVs exhibited expected cup-shaped morphology (Fig. 1c). The EVs size was quantified by a Zetasizer Nano and the mean vesicle diameter was 135 nm (Fig. 1c). Biochemical analysis of EVs showed positive expression of the EVs proteins Alix, CD63, and CD81 (Fig. 1c). We also evaluated the relation between the protein content and the EVs number and found that there are about 10^10^ particles of EVs in 1 µg EVs (Fig. 1d).

### EVs secreted from hiPSC-MSCs ameliorated renal I/R injury

**Fig. 2:**
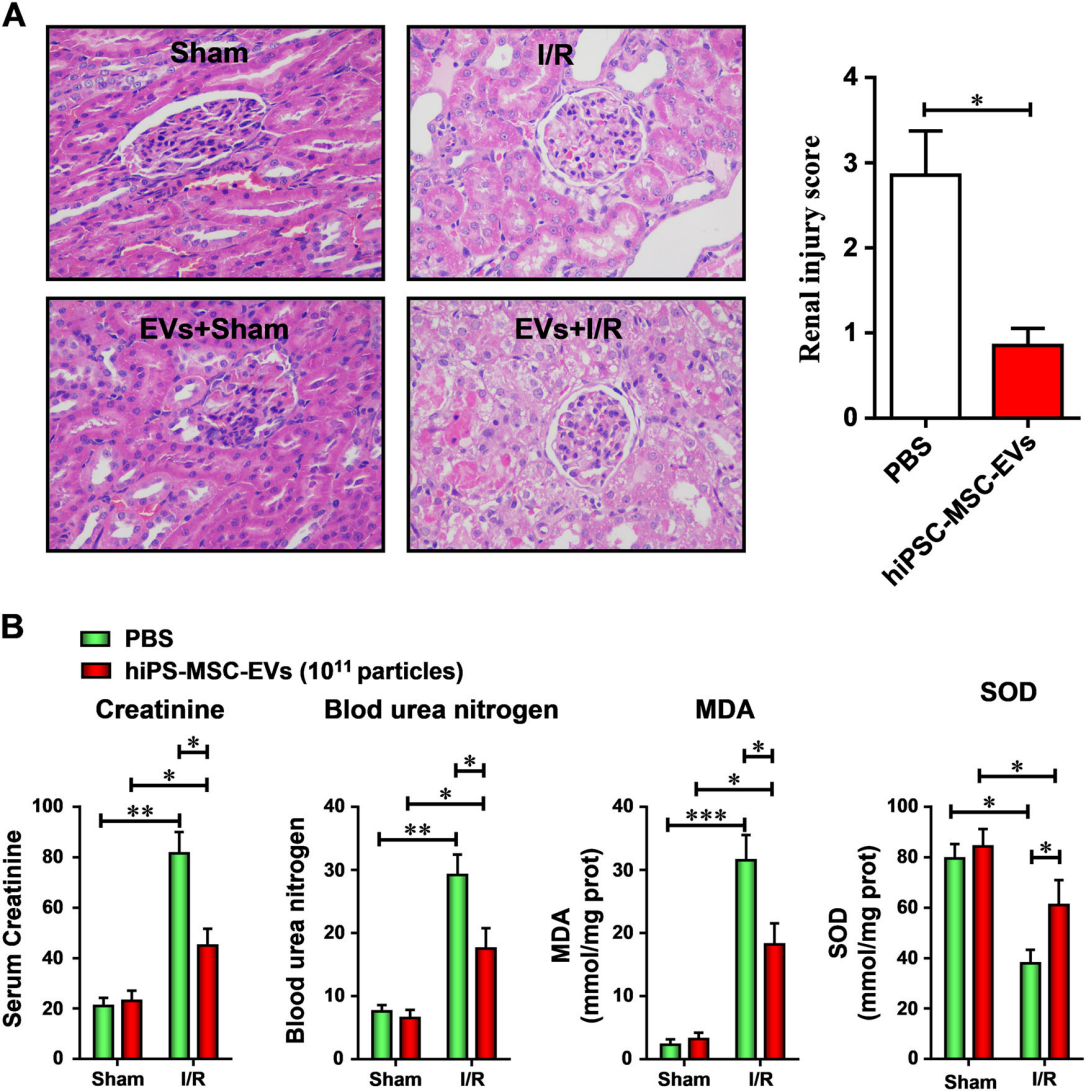
The protective effect of hiPSC-MSCs-derived EVs on renal I/R injury hiPSC-MSCs-derived EVs (10^12^ particles) were suspended in 500 μl PBS (Exo + I/R group) and injected into rats immediately after the initiation of reperfusion via the inferior vena cava. The control group was only injected with 500 μl PBS. **a** Representative histopathologic images of kidney sections harvested at 48 h after reperfusion (H&E staining; scale bars, 100 μm). **b** Blood samples were collected; serum creatinine, blood urea nitrogen, MDA content, and SOD activity were evaluated. Data are presented as means ± SEM, *n* = 6 rats per group; statistical significance: *p < 0.05; **p < 0.01, ***p < 0.001

To evaluate the effect of hiPSC-MSCs-derived EVs on renal I/R injury, EVs were injected into the rat I/R injury model. Compared with control (phosphate-buffered saline (PBS) alone injection), administration of hiPSC-MSCs-EVs (intravenous administration of 10^12^ particles at 1 h before IR) significantly improved the kidney health. Pathologists scored the histology of these kidneys were based on reflecting the grading of tubular necrosis, loss of brush border, cast formation, and tubular dilatation in 10 randomly chosen, non-overlapping fields and in a single-blinded way. The hiPSC-MSCs-EVs-treated group showed lower scores than the corresponding control group (Fig. 2a). By 48 h after the IR procedure, the serum levels of creatinine (Fig. 2b) and blood urea nitrogen (BUN) (Fig. 2c), two indices of renal dysfunction, were markedly increased in the IR group and significantly decreased in the Exo+I/R group. Reoxygenation following ischemia causes tissue oxidative stress, which is considered an important contributor to IRI. Following ischemia–reperfusion, malondialdehyde (MDA) content (Fig. 2d) was greatly increased, and superoxide dismutase (SOD) activity (Fig. 2e) was greatly decreased in kidneys, indicating elevated levels of oxidative stress. However, administration of hiPSC-MSCs-EVs dramatically attenuated the increased MDA content and decreased SOD activity. Collectively, these results demonstrate that hiPSC-MSCs-EVs treatment could ameliorate renal I/R injury.

### hiPSC-MSCs-EVs inhibited necroptosis in renal tubular cells, HK-2 cells during hypoxia/reoxygenation injury

**Fig. 3:**
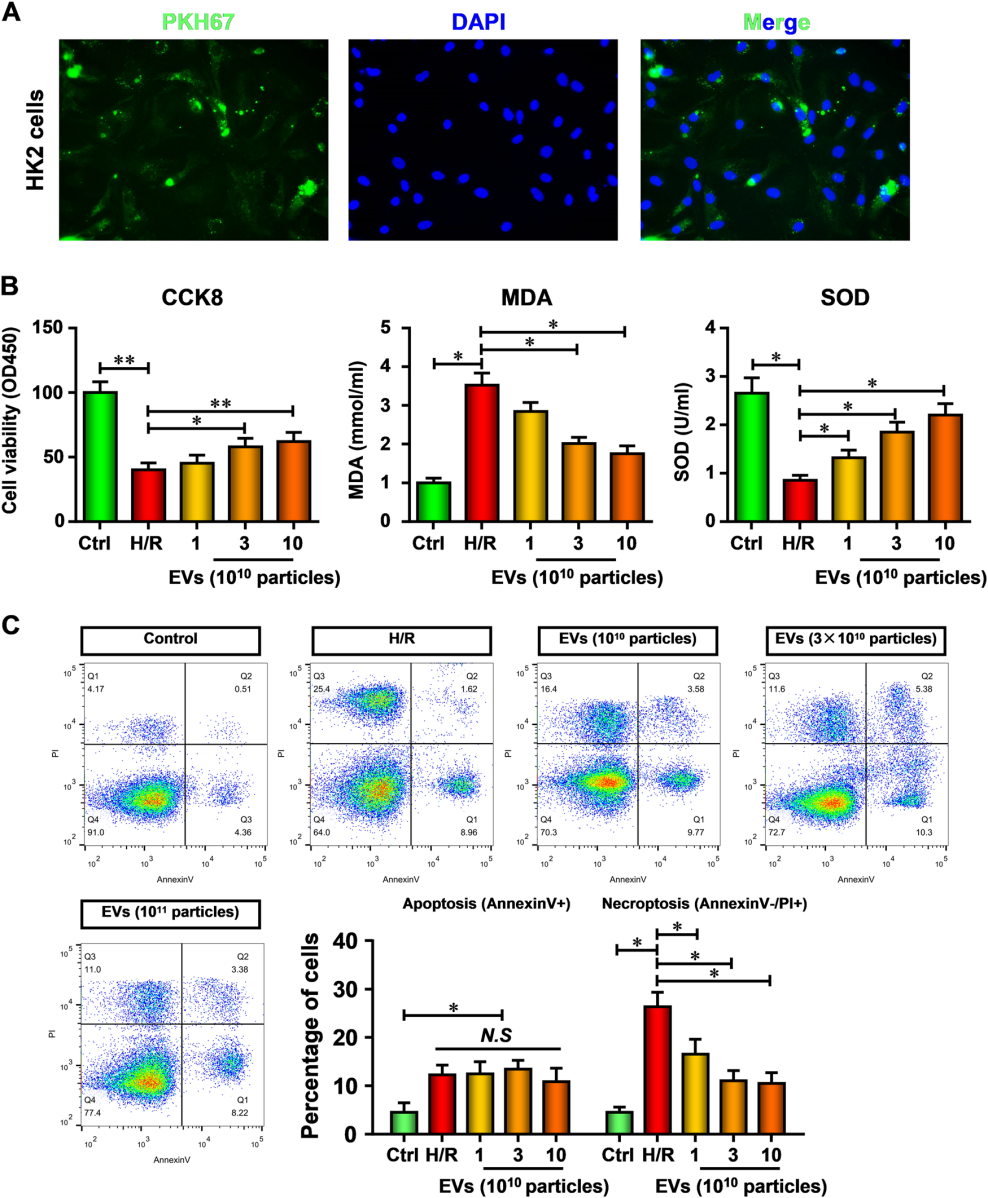
hiPSC-MSCs-EVs protect HK-2 cells against H/R injury via inhibition necroptosis **a** hiPSC-MSCs-derived EVs were fluorescently labeled with a cell membrane marker PKH67 prior to incubation with HK-2 cells. After incubation for 24 h, cells were washed and counterstained with DAPI. The pictures show the representative results from three independent experiments. **b** HK-2 cells were pre-treated with hiPSC-MSCs-EVs (10^11^, 3 × 10^11^, 10^12^ particles) and then subjected to H/R treatment. Cell viability, MDA content, and SOD activity of the HK-2 cells were analyzed. **c** Flow cytometric analysis was used to evaluate the function of hiPSC-MSCs on apoptosis and necroptosis in I/R-injured HK-2 cells. Data are presented as means ± SEM; the results were from three independent experiments; statistical significance: *p < 0.05; **p < 0.01, ***p < 0.001

We next assessed whether the protection by hiPSC-MSCs-EVs was due to inhibition of apoptosis or necroptosis. Firstly, we examined the transfer of EVs components with HK-2 cells. The EVs were labeled with a cell membrane marker PKH67 prior to incubation with HK-2 cells. The results showed that EVs membranes were directly incorporated into the HK-2 cell plasma membrane (Fig. 3a). To determine the effect of hiPSC-MSCs-EVs during hypoxia/reoxygenation (H/R) injury, HK-2 cells were pre-treated with hiPSC-MSCs-EVs (10^11^, 3 × 10^11^, 10^12^ particles) and then subjected to H/R treatment. As shown in Fig. 3b, compared with that of control cells, cell proliferation was markedly inhibited after H/R treatment (*p* < 0.05). Consistently, in cultured HK-2 cells, H/R treatment increased the MDA content (*p* < 0.05) but decreased SOD activity (*p *< 0.05). However, hiPSC-MSCs-EVs dose-dependently preserved the cell proliferation and SOD activity against H/R injury also decreased the MDA content.

Annexin V/PI positivity is an established readout for both apoptotic and necroptotic cell death and allows distinguishing programmed cell death between apoptosis and necroptosis. Flow cytometric analysis showed a markedly increase in apoptotic and necrotic cells (Figure 3c). However, hiPSC-MSCs-EVs treatment dramatically ameliorates the H/R elevated necroptosis, but not apoptosis. Moreover, the protective effect of 3 × 10^11^ and 10^12^ particles of hiPSC-MSCs-EVs seems the same. Taken together, these results provide the first evidence that inhibited necroptosis in kidney cells is critical in the protective effect of hiPSC-MSCs-EVs against renal I/R injury.

### hiPSC-MSCs-EVs directly deliver SP1 into HK-2 cells, transcriptionally increase SK expression and (^3^H) sphinganine-1-phosphate (S1P) formation

**Fig. 4:**
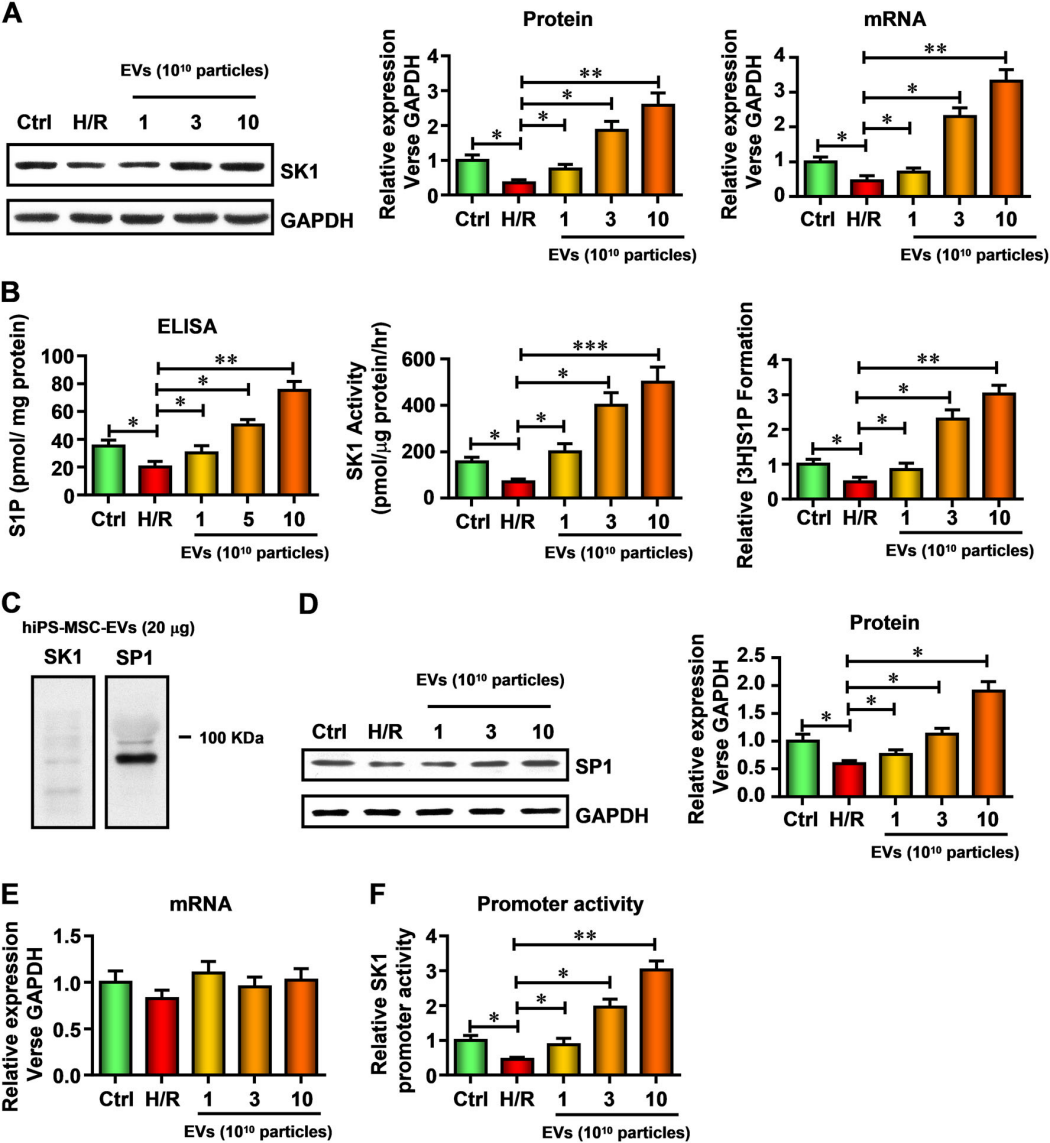
hiPSC-MSCs-EVs directly deliver SP1 into HK-2 cells, which may contribute to the increased sphingosine kinase (SK) expression and (3 H) sphinganine-1-phosphate (S1P) formation in HK-2 cells **a** The relative SK1 expression in HK-2 cells was measured by western blot and real-time PCR and normalized to GAPDH. **b** The relative S1P content, SK activity, and cellular (^3^H) S1P formation in HK-2 cells were measured at 24 h reperfusion. **c** Western blot analysis of hiPSC-MSCs-EVs shows expression of SP1 protein. **d** The relative SP1 protein expression in HK-2 cells was measured by western blot. **e** The relative SP1 mRNA expression in HK-2 cells was measured by real-time PCR. **f** The relative SK1 promoter activity in HK-2 cells was evaluated by luciferase assay. Data are presented as means ± SEM; the results were from three independent experiments; statistical significance: *p < 0.05; **p < 0.01, ***p < 0.001

We have previously reported that exosomes secreted from hiPSC-MSCs-EVs could protect hepatocytes against hepatic I/R injury via activating SK and S1P-dependent pathway^38^. To investigate whether this pathway is also involved in renal protection, we firstly measured the mRNA and protein level changes of SK1 after hiPSC-MSCs-EVs treatment in HK-2 cells, and found a marked increase in SK1 protein levels in a dose-dependent manner (Figure 4a). Then S1P content, SK activity, and (^3^H) S1P formation were measured in H/R-injured HK-2 cells treated with hiPSC-MSCs-EVs. The results showed that after hiPSC-MSCs-EVs treatment for 48 h, S1P content, SK activity, and (^3^H) S1P formation were dose-dependently increased (Fig. 4b).

To further investigate the mechanism by which hiPSC-MSCs-EVs induce increase SK activity, the content of hiPSC-MSCs-EVs was analyzed and found that EVs contained abundant amounts of specificity protein 1 (SP1), a potential transcript factor of SK1, but no detectable SK1 (Figure 4c). Furthermore, the level of SP1 in HK-2 cells was evaluated by western blot (Fig. 4d). The protein level of SP1 in HK-2 cells was significantly decreased, but dose-dependently increased after hiPSC-MSCs-EVs treatment. However, the mRNA level of SP1 was unchanged (Fig. 4e). This suggested that hiPSC-MSCs-EVs may protect HK-2 cells through directly delivering SP1 into HK-2 cells.

**Fig. 5:**
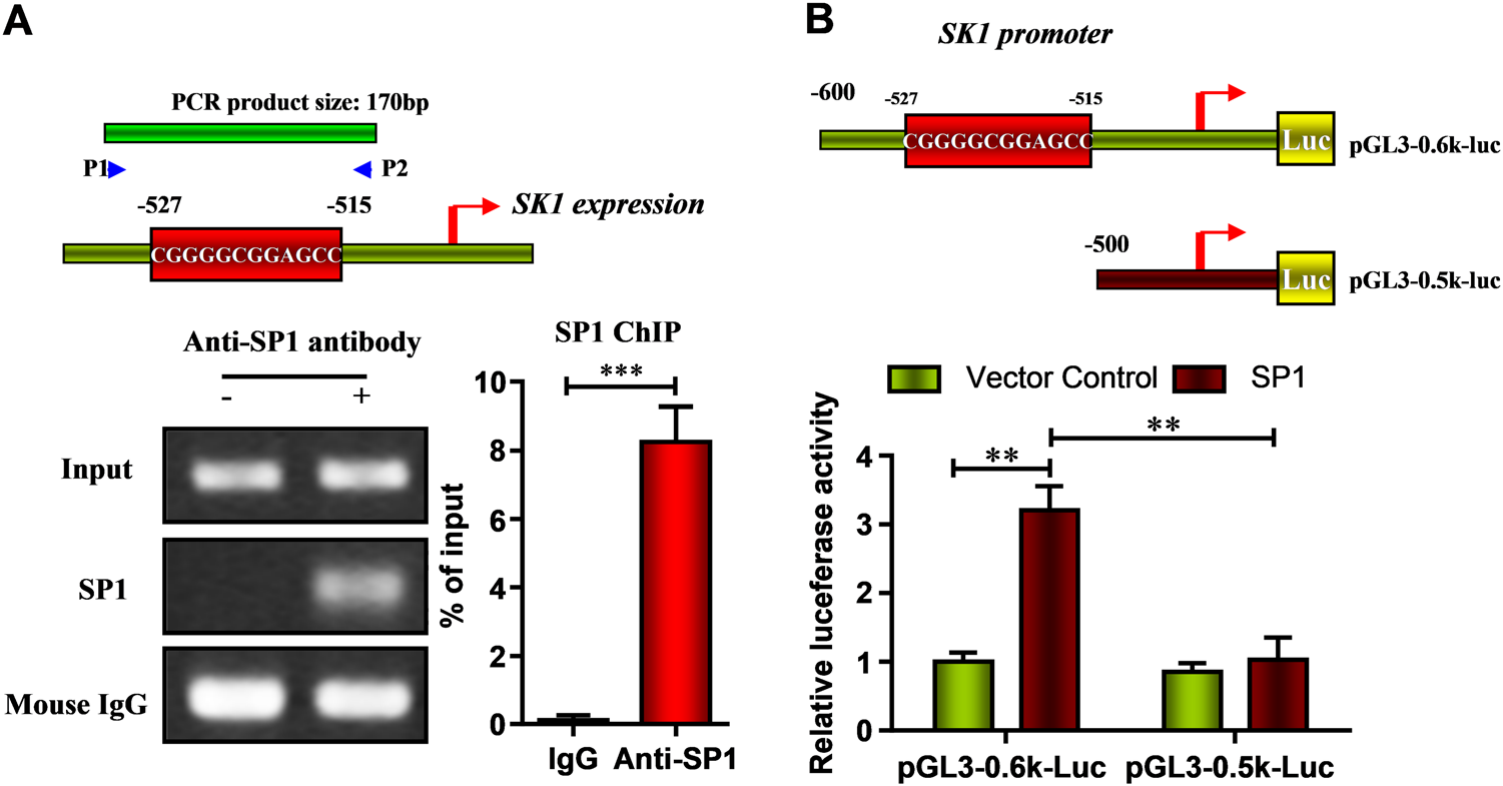
SP1 transcriptionally increased SK1 expression in HK-2 cells **a** Schematic diagrams of the promoter region of SK1 gene indicating the predicted binding sites. A chromatin immunoprecipitation assay (ChIP) was performed and indicated that SP1 interacted with the binding sequence of the SK1 promoter in HK-2 cells. **b** HK-2 cells were transiently co-transfected with empty or SP1 overexpression vectors and with luciferase reporter constructs containing ~0.5 and ~0.6 kb of the SK1 promoter with or without the predicted binding site. Luciferase activity was compared to the control. Data are presented as means ± SEM; the results were from three independent experiments; statistical significance: *p < 0.05; **p < 0.01, ***p < 0.001

We next accessed the promoter activity of SK1 and found a similar increase with the protein level (Figure 4f). We strongly hypothesized that SP1 promoted SK1 gene expression through a direct interaction with the ~0.5–0.6 kb upstream of the SK1 gene promoter. A chromatin immunoprecipitation (ChIP) assay was performed. As illustrated in Fig. 4a, we found specific binding of SP1 to the ~0.5–0.6 kb region of the SK1 promoter, which contained the binding sequence. These data indicate that SP1 interacted with the binding sequence of the SK1 promoter in HK-2 cells. We also used a luciferase reporter construct containing the SK1 promoter with the ~0.6 kb region to monitor SK1 transcription. Overexpression of SP1 caused statistically significant increase in the SK1 promoter activity compared to the empty vector control. In contrast, overexpression of SP1 had no statistically significant effect on the pGL3–0.5 kb-luc construct, which did not contain the binding sequence (Fig. 5b). These results suggest that hiPSC-MSCs-EVs directly delivery SP1 into HK-2 cells, subsequently increase SK expression and improve (3H) sphinganine-1-phosphate (S1P) formation.

### Generation of SP1 knockout hiPSC cell line and hiPSC-MSCs-EVs

**Fig. 6:**
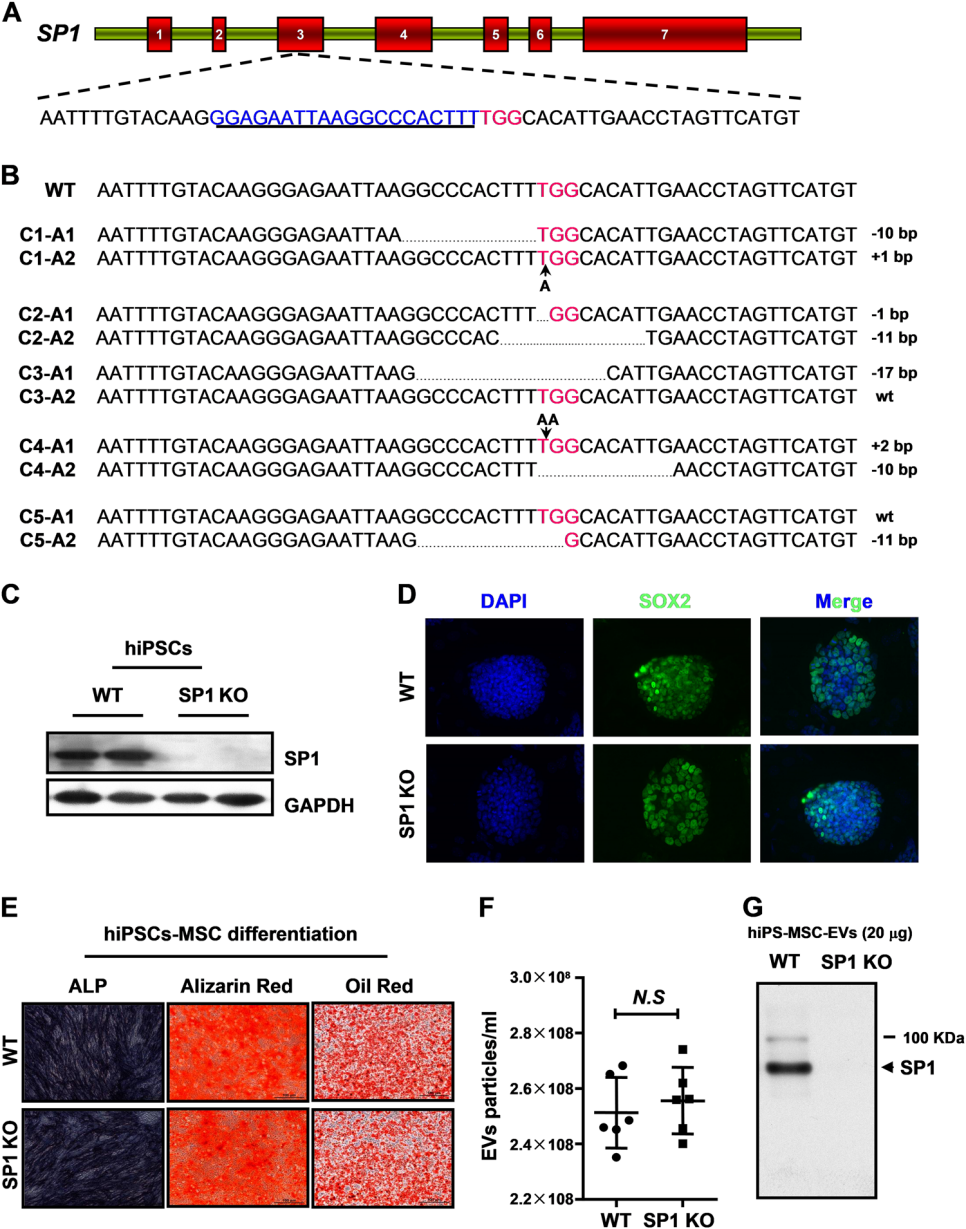
Generation of SP1 knockout hiPSC cell line and hiPSC-MSCs-EVs **a** From the genomic SP1 sequence the target locus for CRISPR editing was chosen and screened by http://crispr.mit.edu for sgRNA binding sites. **b** Sequence alignment of exon 3 from five hiPSC clones. Except clone 3 allele 2 and clone 5 allele 1, all the other alleles were identified contained deletions or insertions that created nonfunctional gene products. **c** Western blot analysis of hiPSC shows undetectable expression of SP1 protein in SP1 KO hiPSC cells. **d** Morphology of hiPSCs was unchanged at SP knockout by immunofluorescence staining. **e** Adipogenic and osteogenetic inductions on wild type and SP1 knockout hiPSCs-MSCs were used to evaluate the multipotential ability by differentiating into cells staining positive with alizarin red and oil red O, respectively. **f** The number of released EVs were measured in culture medium between control and SP1 konckout hsPSC-MSCs cells. **g** The SP1 expression was analyzed in SP1 knockout hiPSCs-MSCs-Exo by western blotting with SP1-specific antibody. Data are presented as means ± SEM; the results were from three independent experiments; statistical significance: *p < 0.05; **p < 0.01, ***p < 0.001

To further confirm the hypothesis that SP1 activated SK1–S1P pathway is critical for the protective effect of hiPSC-MSCs-EVs, we generated an SP1 knockout hiPSC cell line via the CRISPR/Cas system. We first set out to identify an appropriate target site in SP11 for gene editing. The site was selected from a bioinformatic database of 20-bp target-protospacer adjacent motif (PAM) sequences in the human exome that was filtered to minimize off-target cross-reactivity. From a list of several candidates, we chose a target sequence of the human SP1 cDNA (Fig. 6a). Once identified, an oligo pair containing the guide sequence was cloned into pX330, a bicistronic vector that contains the Cas9 enzyme and the programmable chimeric sgRNA^39^. We transfected the px330-SP1 sgRNA and a plasmid encoding GFP into hiPSC cells and isolated the transfected cells by fluorescence-activated cell sorting (FACS). The sorted cells were then plated as individual clones in 96-well plates and expanded. Five clones were probed; exon 3 was PCR amplified from genomic DNA extracted for further sequencing. Sequence analysis of amplicons revealed a mixed population of mutated alleles. We detect one wild-type alleles in clones 3 and 5, while in the other three clones, all of the genomic DNA sequences that were identified contained deletions or insertions that created nonfunctional gene products (Fig. 6b). Taken together with the complete lack of detectable SP1 protein expression by western blotting (Fig. 6c) with SP1-specific antibody, these findings confirm the generation of two SP1 knockout cell lines using the CRISPR/Cas9 system.

We selected clone two hiPSC cells for the further experiments. Immunofluorescence staining assessed the surface antigens of hiPSCs (SOX2) before induction and showed no difference between control and SP1 knockout hiPSC cells (Fig. 6d). Moreover, upon analyzing the effect of adipogenic and osteogenetic inductions on hiPSCs-MSCs, SP1 knockout hiPSC-MSCs fulfill a similarly multipotential role by differentiating into cells staining positive with alizarin red and oil red O, respectively (Fig. 6e). To test whether SP1 knockout affect the release of EVs from hiPSCs-MSCs, we measured the number of EVs in culture medium between control and SP1 knockout hiPSC-MSCs cells (Fig. 6f). At last, the SP1 expression was completely undetectable in SP1 knockout hiPSCs-MSCs-Exo by western blotting with SP1-specific antibody (Fig. 6g).

### SP1–SK1–S1P signaling pathway is critical to the anti-necroptosis effect of hiPSC-MSCs-EVs on H/R-injured HK-2 cells in vitro

**Fig. 7:**
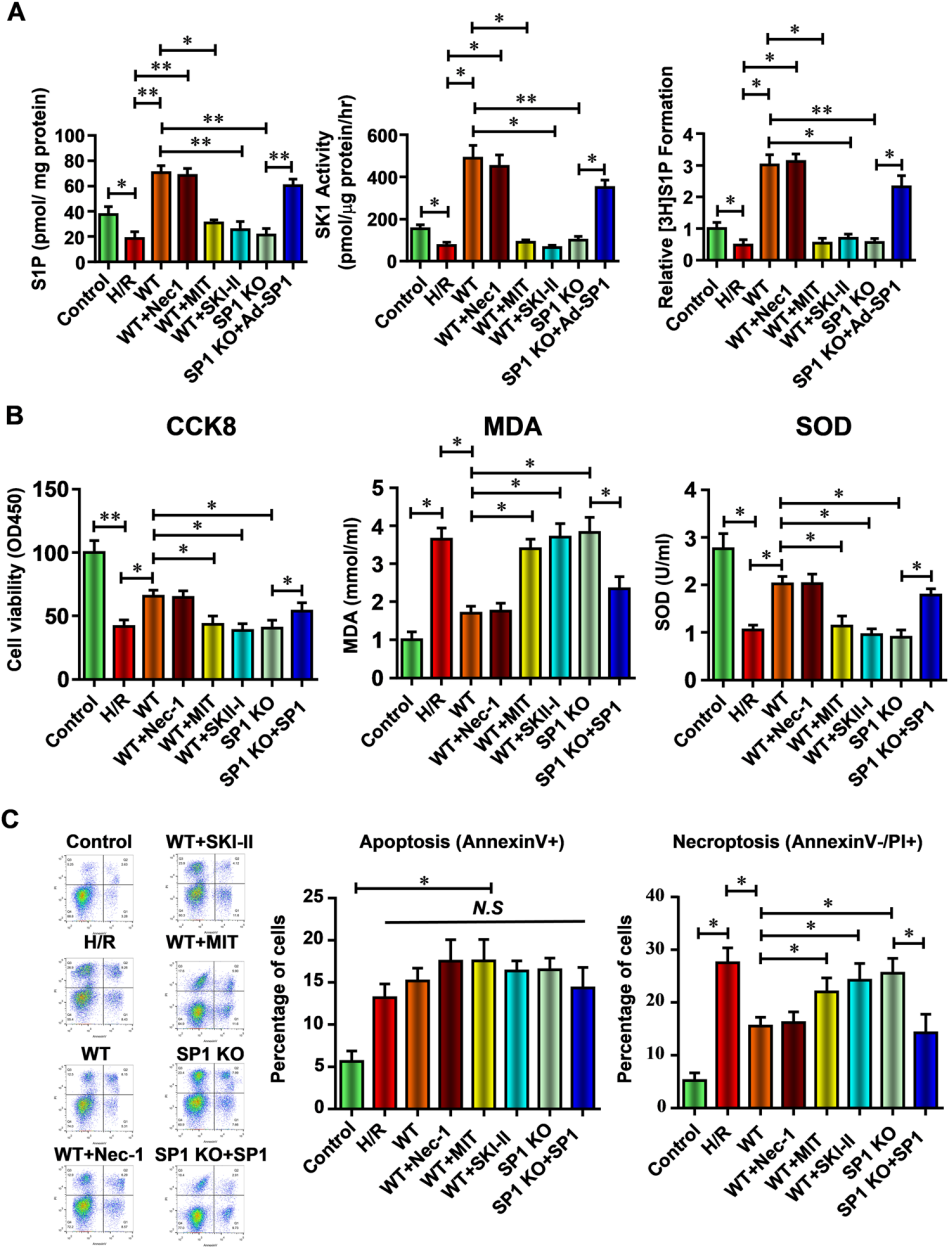
SP1–SK1–S1P signaling pathway is critical to the anti-necroptosis effect of hiPSC-MSCs-EVs against renal I/R injury in vitro **a** With or without the treatment of wild type or SP1 KO hiPSC-MSCs-EVs, Nec-1, MIT, SKI-II, or Ad-SP1, the relative S1P content, SK activity, and cellular (^3^H) S1P formation in HK-2 cells were measured at 24 h reperfusion. **b** Cell viability, MDA content, and SOD activity of the HK-2 cells were analyzed. **c** The function of SP1 KO hiPSC-MSCs, Nec-1, MIT, SKI-II, or Ad-SP1 on the level of apoptosis and necroptosis in I/R-injured HK-2 cells were evaluated by flow cytometric analysis. Statistical significance: *p < 0.05; **p < 0.01, ***p < 0.001

To further demonstrate the hypothesis that SP1 activated SK1–S1P pathway is critical for the renal protective effect of hiPSC-MSCs-EVs, wild type or SP1 knockout hiPSC-MSCs-EVs, combined with Nec-1 (necrostatin-1, a necroptosis inhibitor^40^, 10 μM), MIT (Mithramycin A, an SP1 inhibitor^41^, binding to GC-rich DNA and displacing SP1 transcription factor, 10 μM), or SKI-II (an SK inhibitor^42^, 5 μM) was given to HK-2 cell that underwent H/R injury. As shown in Fig. 7a, the S1P content, SK activity, and (^3^H) S1P formation were parallel in H/R-injured HK-2 cells treated with wild-type hiPSC-MSCs-EVs (WT) with or without Nec-1 treatment. After SP1 inhibition with MIT or SK inhibition with SKI-II in HK-2 cells, hiPSC-MSCs-EVs failed to increase the S1P content, SK activity, and (^3^H) S1P formation in H/R-injured HK-2 cells (Fig. 7a). EVs from SP1 knockout hiPSC-MSCs also cannot increase the level of S1P content, SK activity, and (^3^H) S1P formation (Fig. 7a). However, adenovirus-mediated overexpression of SP1 in SP1 knockout hiPSC-MSC cells restored the promoting effect of secreted EVs on S1P content, SK activity, and (^3^H) S1P formation (Fig. 7a). These results suggested the specific role of SP1 in the hiPSC-MSCs-EVs on the activation of SK1–S1P pathway.

Similarly, Nec-1 failed to improve the protective effect of wild-type hiPSC-MSCs-EVs on decreased cell proliferation, increased MDA content, and decreased SOD activity (Fig. 7b). After SP1 inhibition with MIT or SK inhibition with SKI-II in HK-2 cells, hiPSC-MSCs-EVs showed no effect on the cell proliferation, MDA content, and SOD activity in H/R-injured HK-2 cells (Fig. 7b). EVs from SP1 knockout hiPSC-MSCs also failed to preserve these parameters (Fig. 7b), yet adenovirus-mediated overexpression of SP1 in SP1 knockout hiPSC-MSC cells restored the protective effect of secreted EVs on these parameters of cell viability (Fig. 7b). These results suggested the specific role of SP1 activated SK1–S1P pathway on the protective effect against H/R injury.

At last, Nec-1 unable to further inhibit necroptosis during H/R injury in hiPSC-MSCs-EVs-treated HK-2 cells (Fig. 7c). Meanwhile, both SP1 and SKI-II inhibition completely abolished the anti-necroptosis effect of hiPSC-MSCs-EVs (Fig. 7c). After SP1 knockout in hiPSC-MSCs, the secreted EVs can no longer decrease the H/R-induced necroptosis in HK-2 cells unless restoring the level of SP1 by Ad-SP1 (Fig. 7c). These data prove that hiPSC-MSCs-EVs deliver SP1 to the target HK-2 cells and result in intracellular activation of SK1 expression and generation of S1P, which inhibit H/R-induced necroptosis.

### SP1–SK1–S1P signaling pathway is critical to the anti-necroptosis effect of hiPSC-MSCs-EVs against renal I/R injury in vivo

**Fig. 8:**
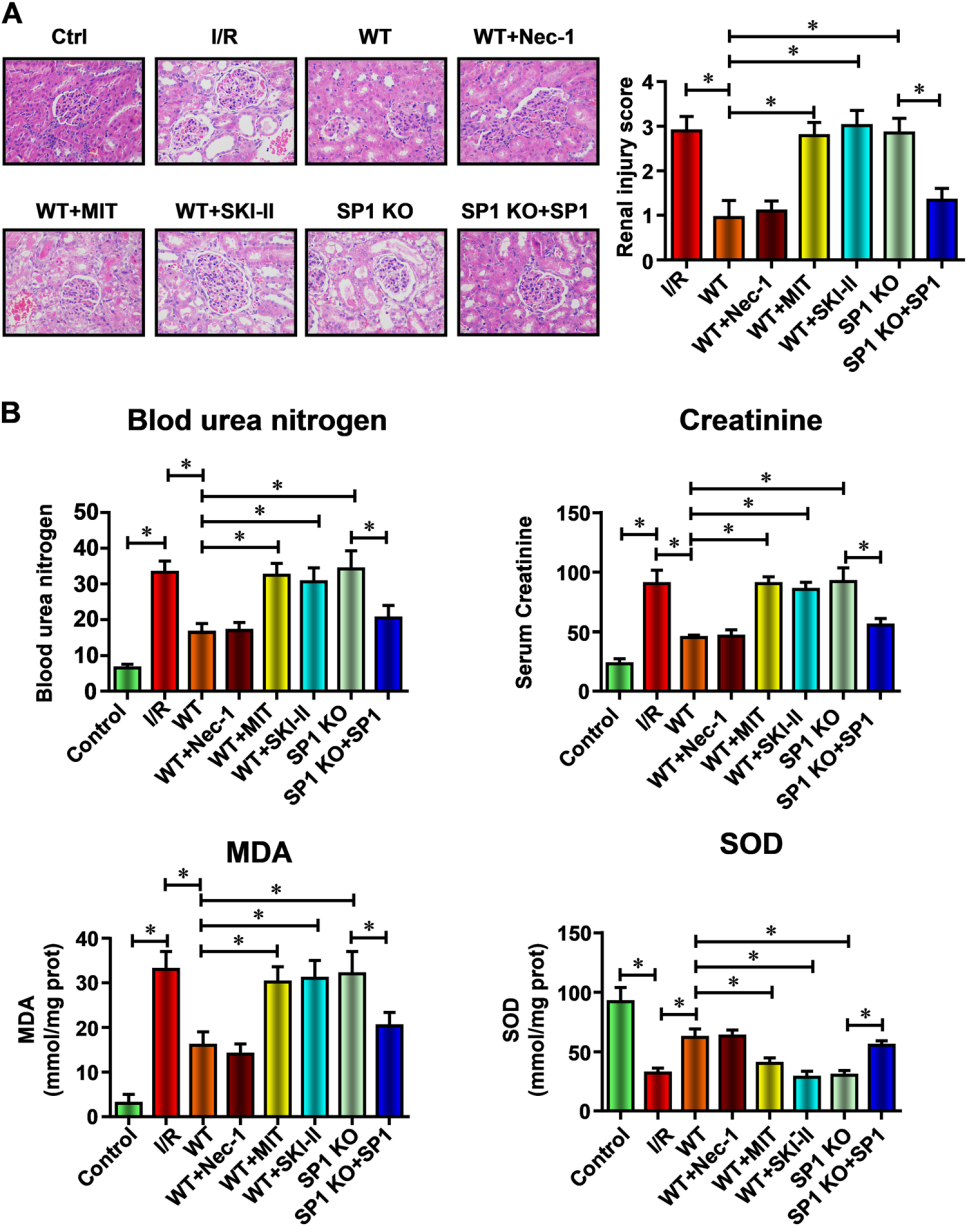
SP1–SK1–S1P signaling pathway is critical to the anti-necroptosis effect of hiPSC-MSCs-EVs against renal I/R injury in vivo **a** Representative histopathologic images of kidney sections harvested at 48 h after reperfusion (H&E staining; scale bars, 100 μm). **b** Blood samples were collected, serum creatinine, blood urea nitrogen, MDA content, and SOD activity were evaluated. Data are presented as means ± SEM, *n* = 6 mice per group; statistical significance: *p < 0.05; **p < 0.01, ***p < 0.001

To demonstrate the specific role of SP1 activated SK1–S1P pathway in the renal protective effect of hiPSC-MSCs-EVs in vivo, rats were injected with hiPSC-MSCs-EVs. As expected, treatment of rats with EVs from wild-type hiPSC-MSCs-EVs (WT) resulted in significant renal protections with the decreased pathologists score (Fig. 8a), creatinine, MDA content, BUN, and the preserved SOD activity (Fig. 8b). Next, the necroptosis inhibitor Nec-1 (100 mg/kg), SP1 inhibitor MIT (60 mg/kg), or SK inhibitor SKI-II (50 mg/kg) was subcutaneously administered to rats at 15 min before the administration of wild-type hiPSC-MSCs-EVs (WT). As expected, pre-treatment of Nec-1 (WT+Nec-1) showed no difference with the administration of wild-type hiPSC-MSCs-EVs only, which suggested that hiPSC-MSCs-EVs mainly protect the renal cell via inhibition necroptosis. However, SP1 inhibition (WT+MIT) or SK1 inhibition (WT+SKI-II) completely abolished the renal protective effect of hiPSC-MSCs-EVs in a rat I/R injury model, demonstrated by changes in histopathologic score (Fig. 8a), the serum levels of creatinine, MDA content and BUN, and the SOD activity (Fig. 8b). We then isolated EVs from hiPSC-derived MSCs in which SP1 had been knocked out by the CRISPR/Cas9 system (SP1 KO). Like previous in vitro experiments, SP1 KO EVs failed to diminish the renal injury, while SP1 overexpression in SP1 knockout hiPSC-MSCs by Ad-SP1 (SP1 KO+Ad-SP1) regained the renal protective effect of secreted EVs. Taken together, these results prove that hiPSC-MSCs-EVs deliver SP1 to the target renal cells resulting in intracellular activation of SK1 expression and generation of S1P which inhibits I/R induce necroptosis in vivo.

## Discussion

In this paper, we describe a novel and protection function of hiPSC-MSCs secreted EVs against renal I/R injury. Our results show that the EVs released by hiPSC-MSCs can protect renal cells against I/R-induced necroptosis in a rat model. Mechanistically, the hiPSC-MSCs-EVs can directly delivery SP1 into renal cells and inhibit necroptosis via transcriptional activation of SK1 expression and generation of S1P. These findings largely extend our current understanding of hiPSC-MSC-derived EVs, and suggest that they may function to restore renal function after injury, dysfunction, or disease. Furthermore, our data suggest that hiPSC-MSC-derived EVs represent a novel therapeutic approach for kidney diseases.

Recently, MSCs injection resulted in lower pro-inflammatory and apoptotic scores, and higher anti-inflammatory and mitogenic indices, thus effectively ameliorating experimental AKI^43–50^. Because this positive functional effect was accompanied by rapid disappearance of administered MSCs from the kidney, it is supposed that these cells act principally through paracrine mechanisms, especially MSCs-derived EVs^51–55^. Due to the commonly used adult MSCs derived from tissues have limited abilities in proliferation, an alternate source of MSCs is in need. The hiPSC-derived MSCs was found to be a potential source of MSCs because the cells exhibited better survival, proliferation, and differentiations potentials^20,21,35,56^. However, the direct use of hiPSCs-MSCs is limited due to the problems like potential immunological rejection, chromosomal variation, and so on. As EVs are considered to be an important mediator of hiPSCs-MSCs, the therapeutic effects of hiPSCs-MSCs-derived EVs have also been investigated extensively in various disease models, and the results revealed that they could preserve the function of myocardium and the hepatic function after I/R injury^24,36^. Our current data reveal that hiPSCs-MSCs-derived EVs may represent a novel highly significant function to protect tissues against I/R injury. The fact that EVs from SP1 knocked out hiPSCs-MSCs were unable to induce renal protection demonstrates a specific characteristic of hiPSCs-MSCs-Exo.

Our results demonstrate that the renal protective effects of hiPSC-MSCs-EVs are mediated by activated SK1 expression and increased S1P formation. Importantly, our results suggest that hiPSC-MSCs-EVs can fuse with renal cells and delivery SP1 into target cells, subsequently and transcriptionally active SK1 expression and increase S1P formation. S1P has been shown to function as both an extracellular ligand for specific G protein-coupled receptors (GPCRs) as well as an intracellular second messenger in promoting cell growth and survival^57,58^. Some other studies have found that activation of SK, the enzyme catalyzing the formation of S1P from its precursor sphingosine, with a resultant increase in levels of S1P, and activation of S1P receptors, which have been shown to be involved in protection from IR injury in the heart^42,57,58^, liver^59^, and kidney^60,61^. In this study, we initially found that the SP1–SK1–S1P signaling pathway triggers the renal protective effects via inhibiting necroptosis but nor apoptosis. The necroptosis inhibitor Nec-1 fails to further improve renal protective effects of hiPSC-MSCs-EVs. Undoubtedly, necroptosis and apoptosis coexist in the pathophysiological process of AKI^17,62^. However, whether necroptosis is relevant to the damage of kidney function during AKI is challenged recently. Despite some limitations in the detection techniques for apoptosis,^62^ the effectiveness of anti-apoptosis therapeutic interventions in previous studies have proven the contribution of apoptosis to AKI^62^, which should not be neglected even from current perspective. In fact, necroptosis, apoptosis, and other modes of regulated cell death orchestrate the pathogenesis of AKI together; and the relative contribution of each cell death to AKI depends on the type and severity of the injury. In the present study, we initially illustrate the certain contribution of necroptosis to the renal dysfunction in AKI, and suppose that blocking necroptosis in conjunction with current clinical treatment can be applied to improve the therapeutic efficacy for renal diseases.

**Fig. 9:**
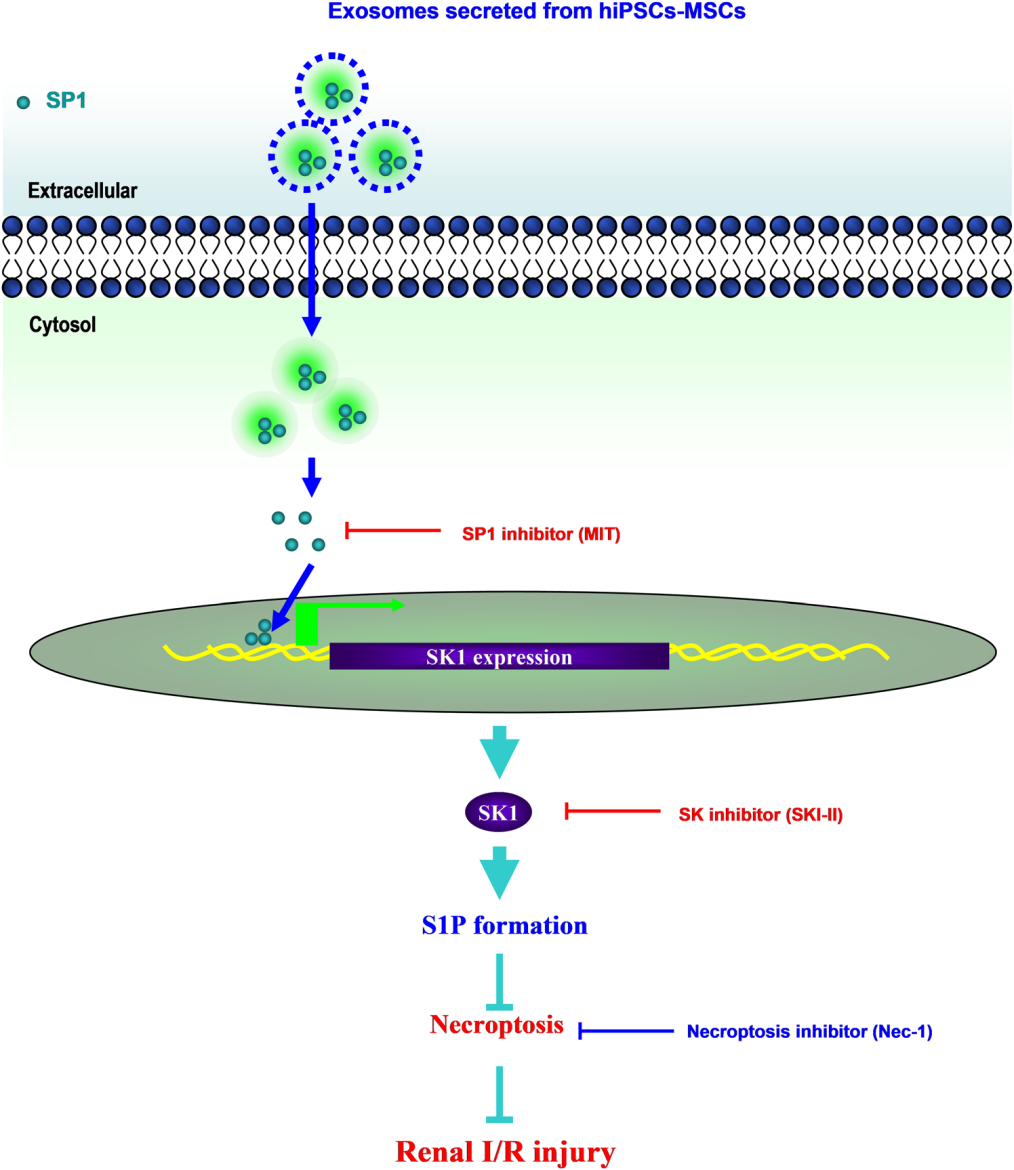
Schematic diagram of the proposed cellular mechanisms for the protection of hiPSC-MSCs-derived EVs after renal I/R injury Based on our data, we propose that hiPSC-MSCs-EVs alleviate renal I/R injury by delivering SP1 to the target renal cells and resulting in intracellular activation of SK1 expression and generation of S1P, by which inhibits I/R-induced necroptosis

In conclusion, we are the first to describe the anti-necroptosis effect of hiPSC-MSCs-derived EVs against renal I/R injury, both in vitro and in vivo. Furthermore, we investigated the molecular mechanisms and found that EVs could delivery SP1 into target renal cells and transcriptionally activate the expression of SK1 and the generation of S1P (Fig. 9). These findings suggest a novel mechanism for renal protection against I/R injury, and indicate a potential therapeutic approach for a variety of renal diseases and renal transplantation.

## Materials and methods

### Animals and reagents

Male Sprague-Dawley rats (4–5 weeks of age) weighing 180–220 g were obtained from the Slac Laboratory Animal Center (Shanghai Slac Laboratory Animal Co. Ltd, China). The experimental protocols were approved by the Institutional Animal Care and Use Committee of the Transplantation Center of Ren Ji Hospital, School of Medicine, Shanghai Jiao Tong University. Necroptosis inhibitor (Nec-1, necrostatin-1), Sphingosine kinase inhibitor (SKI-II), and SP1 inhibitor (MIT, Mithramycin A) were purchased from Sigma Aldrich (St. Louis, MO).

### Renal I/R model

A rat model of renal IRI was established as previously described^63^. Briefly, rats were fasted overnight, anesthetized by an intraperitoneal injection of 3% pentobarbital sodium (0.1 ml/100 g body weight), and subjected to an abdominal incision. Their rectal temperatures were maintained at 37 °C using a thermistor connected to a servo-controlled heating pad (model D1-L; Haake, Tokyo, Japan). The renal pedicles of the IRI group of rats were dissected and clamped with non-traumatic clamps for 30 min. The renal pedicles were then reperfused in situ for 48 h, after which six rats from each group at each time point were euthanized by decapitation, and their renal tissues were carefully dissected for subsequent experiments. Sham-operated control rats were subjected to an abdominal incision but did not undergo clamping of the renal pedicles. Blood samples were collected from individual rats at each time point after reperfusion to measure concentrations of serum creatinine and BUN by an automatic biochemistry analyzer (Hitachi 7060).

### Measurement of MDA content and SOD activity

Oxidative stress in the kidneys and HK-2 cells was assessed by measuring the MDA content and SOD activity. MDA is a terminal product of lipid peroxidation; its intracellular concentration was determined with commercial kits (Beyotime) that use the thiobarbituric acid method to form a red product with a maximum absorbance at 535 nm. The results are reported as μmol per milligram of extracted protein. The SOD activity in the kidneys was detected using a Total Superoxide Dismutase Assay Kit (Beyotime) and reported as U/mg protein.

### Histology

Kidneys were dissected as indicated in each experiment and infused with 4% neutral-buffered formaldehyde, fixated for 48 h, dehydrated in a graded ethanol series and xylene, and finally embedded in paraffin. The sections were stained with hematoxylin and eosin and then analyzed using an Axio Imager microscope (Zeiss, Oberkochen, Germany) at ×400 original magnification. Micrographs were digitalized using an AxioCam MRm Rev. 3 FireWire camera and AxioVision Rel. 4.5 software (Zeiss). Organ damage was quantified by an experienced pathologist in a double-blind manner on a scale ranging from 0 (unaffected tissue) to 5 (severe organ damage).

### hiPSCs-derived MSCs

The iPSCs were cultured in mTESR1 medium (StemCell Technologies) to 90% confluency. Then mTESR1 was replaced by MSC medium (Dulbecco’s modified Eagle's medium (DMEM)-low glucose supplemented with 10% fetal bovine serum (FBS) and 2 mmol/l l-glutamine). After 2 weeks of culturing in MSC medium, cells were trypsinized and expanded in 0.1% gelatin-coated dishes in MSC medium. Cells were passaged until they developed the fibroblast-like morphology. Then these cells were ready to be used in the MSC phenotypic characteristics analysis and further experiments. For the EVs isolation, conventional culture medium was replaced with an exosome-depleted FBS-contained (Exo-FBS-250 A-1; System Biosciences, Mountain View, CA, USA) medium when the cells reached 70–80% confluence, and the MSCs were cultured for an additional 72 h. The media were then collected and EVs were isolated by the ExoQuick exosome isolation method according to the manufacturer’s instruction.

### Immunofluorescence staining

For immunofluorescence staining, cultured hiPSCs and hiPSCs-MSCs were fixed with 4% buffered paraformaldehyde in PBS, and incubated with blocking solution (1% bovine serum albumin (BSA) and 0.1% Triton X in PBS) at room temperature for 2 h. Then cells were incubated with primary antibodies against SOX2 (Abcam) and CD90 (Abcam) diluted in PBS overnight at 4 °C. After that, appropriate secondary antibodies (Abcam) were added and incubated with cells for 1 h at room temperature. The nuclei were stained with 4′,6-diamidino-2-phenylindole (DAPI; Beyotime, Shanghai, China) for 20 min at room temperature. Immunofluorescence was examined under a fluorescence microscope (Nikon 80i, Otawara, Tochigi, Japan).

### Flow cytometry

To analyze the characteristics of cultured hiPSC-MSCs, cells were incubated with 1% BSA for 30 min (Gibco) in PBS to block nonspecific antigens. Conjugated monoclonal antibodies (PE-CD34, PE-CD45, APC-HLA-DR-PE, FITC-CD29, FITC-CD90, and FITC-CD105; Biolegends) were used according to the manufacturer’s instructions. To evaluate the apoptosis level of HK-2 cells, we analyzed the percentage of the early apoptotic cells using an Annexin V-FITC apoptosis detection kit according to the manufacturer’s instructions. The samples were analyzed using an Epics xL flow cytometer (Beckman Coulter, High Wycombe, UK).

### Multipotential of hiPSC-MSCs

Osteogenesis and adipogenesis were examined to determine the multipotential of hiPSC-MSCs. For induction, 1 × 10^5^ hiPSC-MSCs were seeded in 24-well plates until they reached 80% confluency, at which point the medium was replaced with osteogenesis medium or adipogenesis medium (ThermoFisher). The cells were fixed with 4% paraformaldehyde after 21 days and stained in alizarin red or oil red O for observation under a fluorescence microscope (Nikon 80i, Otawara, Tochigi, Japan).

### Isolation of EVs

EVs were collected using Exoquick precipitation. Briefly, cells were grown to 80% confluency and then cultured for 48 h. The condition medium was obtained and filtered by a 0.45-lm PVDF filter (Millipore, Billerica, MA) to further eliminate cellular debris. ExoQuick Precipitation Solution (SBI System Biosciences, Mountain View, CA) was added to the supernatant and mixed well, and then the mixture was precipitated by staying in 4 °C for 30 min. EVs were harvested after centrifuging at 4 °C 1500 *g* for 30 min. EVs pellets were re-suspended with PBS and their total protein concentration was determined by bicinchoninic acid assay (ThermoFisher).

### EVs characterization

EVs morphology was visualized by transmission electron microscopy. Briefly, EVs were diluted to 1 mg/ml with PBS. Then, a specimen of EVs was spotted onto a glow-discharged copper grid on the filter paper and dried for 20 min under infrared lamp. Finally, the grid was stained with 2% uranyl acetate at pH 7.0 for 40 s and air-dried at room temperature. EVs were examined under transmission electron microscopy (H-600 HITACHI microscope, Japan) at 80 keV. The size of the EVs was determined using a Zetasizer Nano (Malvern Instruments, Malvern, UK), and the purity was assessed by western blot with EVs markers (Alix, CD63, and CD81). The number of EVs was assessed by the CD81-antigen ELISA kit (ThermoFisher).

### Cell culture and H/R treatment

The human renal proximal tubular cell line HK-2 was obtained from American Type Culture Collection (Manassas, VA, USA), plated in 100-mm culture dishes, and cultured in Dulbecco’s modified Eagle’s medium (ThermoFisher) supplemented with 10% fetal bovine serum, 100 U/ml penicillin, 2 mM glutamine, 100 μg/ml streptomycin, and 1 mM HEPES buffer. The cultures were incubated at 37 °C in humidified air containing 5% CO_2_. The medium was replaced every other day. For the H/R group, cells were exposed to hypoxia (5% CO_2_, 1% O_2_, and 94% N_2_) for 12 h followed by 24 h of reoxygenation (5% CO_2_, 21% O_2_, and 74% N_2_).

### Adenovirus-mediated overexpression of SP1 in SP1 knockout hiPSC-MSC cells

A recombinant adenovirus encoding human SP1 (Ad-SP1) was constructed and generated by Genelily Biotech, Inc. (Shanghai, China) as previously described using a reverse genetic method. SP1 expression mRNA and protein expression was verified using RT-qPCR and western blot assay. SP1 knockout hiPSC-MSC cells were infected with a multiplicity of infection of 10 purified Ad-SP1.

### Cell viability assay

At the end of the indicated time, HK-2 cells were treated with CCK8 (10 μl/well; Sigma, USA) for an additional 2 h, then we recorded the absorbance at 450 nm using a microplate absorbance reader (Tecan, Safire II, Switzerland).

### EVs-HK-2 fusion

EVs were labeled with 2 μM PKH67 (Sigma Aldrich) for 5 min. After washing, they were incubated with cultured HK-2 cells for 24 h. Then the samples were washed and counterstained with DAPI, followed by analyzing with fluorescence microscopy.

### Quantitative real-time polymerase chain reaction

Total RNA was extracted from rat kidneys with TRIzol reagent (Invitrogen, Shanghai, China) according to the manufacturer’s instructions. Total RNA (1 μg) was transcribed into cDNA by Superscript II reverse transcriptase (Invitrogen) and random primer oligonucleotides (Invitrogen). Real-time PCR was performed with the 7900 HT Real-Time PCR System (Applied Biosystems) for 40 cycles with GAPDH and as an internal control. The following primers were used in the study:

**Table:** 

Gene name	Forward primer (5′–3′)	Reverse primer (5′–3′)
SK1	ATCTCCTTCACGCTGATGC	GTGCAGAGACAGCAGGTTCA
SP1	AGGCACAAACGTACACACAC	TGACGTTGATGCCACTGTTG
GAPDH	GGGAAACTGTGGCGTGAT	GAGTGGGTGTCGCTGTTGA

### ChIP assay

A ChIP assay was performed as described previously. Briefly, cells were crosslinked with formaldehyde, and chromatin was fragmented by sonication. Chromatin was immunoprecipitated with anti-SP1 (Santa Cruz) or control IgG, and purified co-precipitated DNA was quantified by PCR with Ex TaqTM Polymerase (TaKaRa, Otsu, Japan). The PCR products were then analyzed using agarose gel electrophoresis and EtBr staining for visualization. The primers used to amplify the DNA fragments were forward 5′-GGAACCAGCTCGTGGCCCGG-3′ and reverse 5′-GCAGCTCGTCCCAAGCTCAG-3′.

### Luciferase assay

The SK1-luciferase constructs were created by inserting an ~0.5 kb and an ~0.6 kb fragment encompassing the predicted binding site into the pGL4-BASIC-luciferase plasmid (Promega, Tokyo, Japan). The primers used to amplify the DNA fragments were SP1_P1 (forward 5′-GGAACCAGCTCGTGGCCCGG-3′ and reverse 5′-TGCTGGGCACGAAGTTCTGG-3′) and SP1_P2 (forward 5′-AGGCTCAGTGCCCTCCCCGC-3′ and reverse 5′-TGCTGGGCACGAAGTTCTGG-3′). A commercial plasmid containing a CMV-driven Renilla reporter system was used as an internal control (Promega). HK-2 cells were plated in six-well plates at 50–70% confluence and were co-transfected with the pCMV-SP1 construct or with an equimolar amount of the empty pCMV vector and the pGL4-SP1-P1 or pGL4-SP1-P2 construct utilizing Lipofectamine 3000 reagents (ThermoFisher). The media was changed 2 h prior to transfection. After the media was changed, the cells were incubated in 10% DMEM for 24 h. The luciferase assays were performed using the Dual-Luciferase Reporter Assay System according to the manufacturer’s instructions (Promega). Briefly, 100 ml of luciferase substrate was added to 20 ml of lysate, and luciferase activity was measured using an LB940 Multilabel Reader (Berthold Technologies, Bad Wildbad, Germany). Each luciferase assay was performed in triplicate.

### Generation of SP1 knockout hiPSC cell lines

Firstly, a 20-bp guide sequence (5′-GGAGAATTAAGGCCCACTTT-3′) targeting DNA within the third exon of SP1 was selected from a published database of predicted high-specificity PAM target sites in the human exome^64^. Two complementary oligos (5′-CACCGGAGAATTAAGGCCCACTTT-3′ and 5′-AAACAAAGTGGGCCTTAATTCTCC-3′) containing the SP1 guide sequence and BbsI ligation adapters were synthesized by Genelily Biotech. One hundred micromolar of each oligo was annealed using T4 polynucleotide kinase (New England Biolabs) and 1 μl 10× T4 Ligation Buffer in a total volume of 10 μl in a Bio-Rad thermal cycler. The cycling conditions were 37 °C for 30 min, then 95 °C for 5 min, followed by a ramp to 25 °C at 5 °C/min. The annealed oligo was ligated into the BbsI-digested pX330 vector using 5 μl of 2× QuickLigation Buffer and 1 μl of QuickLigase (New England Biolabs). The ligation mixture was treated with Plasmid Safe exonuclease (Epicentre) and transformed in OneShot chemically competent Stbl3 cells (Life Technologies). After plasmid DNA extraction (Qiagen), the sequence of the construct was verified by automated DNA sequence analysis. Secondly, HK-2 cells were cultured in six-well dishes to 70–80% confluence. Cells were co-transfected with 1 μg of SP1 sgRNA plasmid plus 1 μg of pLVX-GFP (Clontech) and 5 μl of Lipofectamine 3000/well. pLVX-GFP-derived green fluorescent protein was used as a fluorescent marker to sort transfected cells. Forty-eight hours post-transfection, cells were pelleted in PBS + 2% FBS and sorted in 96-well plates using FACS with a FACSAria II cell sorter (BD BioSciences). Single cells from the population of GFP-expressing cells were expanded to obtain individual clones. Genomic DNA was isolated from edited clones and nonedited hiPSC cells as described above. Exon 3 of SP1 was PCR amplified using the SP1-specific PCR primers. The PCR products were A-tailed and cloned into pGEM-T Easy (Promega). Individually cloned amplicons were then analyzed by Sanger sequencing. At last, positive clones were lysed in detergent solution for 20 min at 4 °C and centrifuged at 16,000 *g* for 10 min, and 20 μg of supernatant was fractionated on 4–20% sodium dodecyl sulfate–polyacrylamide gel electrophoresis (SDS-PAGE) gels, transferred on to nitrocellulose, and screened by immunoblotting with SP1 antibodies.

### SK activity assay

SK activity was measured as described by Vessey et al.^65^. Briefly, enzyme preparations from HK-2 cells (200 μg total protein) were incubated in assay buffer (0.05% Triton X-100, 250 mM KCl, 0.05 μM (3H) sphingosine (20 Ci/mmol; American Radiolabeled Chemicals), 5 mM ATP, 10 mM MgCl_2_, 100 mM Tris pH 8.0) at 20 °C for 30 min, in a total volume of 100 μl. Different incubation times yielded a linear relationship with SK activity (data not shown). Then, (^3^H)S1P was extracted using 1.2 ml of methanol:chloroform:trisodium EDTA (pH = 9) (1:2:1). After vortexing and centrifuging for 5 min at 2700 *g*, the upper aqueous phase was collected and counted in a liquid scintillation counter (Packard BioScience, Meriden, CT). To verify the product of our extraction, aliquots of the upper aqueous phases were resolved on silica 60 A° TLC plates (Whatman, Florham Park, NJ) using 1-butanol:methanol:acetic acid:ddH_2_0 (80:20:10:20). The lanes were scraped in 1-cm increments and counted in a liquid scintillation counter. We found that the peaks from our extraction had the same relative mobility (Rf) as purified (^3^H) S1P (data not shown).

### Measurement of (^3^H) S1P synthesis in HK-2 cells

Cellular (^3^H) S1P formation in HK-2 cells was measured using a method described by Lavieu et al.^66^. Confluent cells in six-well plates were cultured in serum-free media containing 0.02 μM (^3^H) sphingosine (20 Ci/mmol) overnight to allow for equilibration. Then the media were removed and the cells were scraped in 100 μl lysis buffer. (^3^H) S1P was extracted from the cell lysates as described above and counted.

### Western blot

Tissues or cells were lysed in ice-cold RIPA lysis buffer (Solarbio, Beijing, China) containing 0.1 mM PMSF and protease inhibitor (Roche) for 30 min on ice. Samples were subjected to 12% SDS-PAGE and transferred to nitrocellulose membranes. Membranes were probed with the following primary antibodies: anti-Alix (Abcam, Cambridge, UK), anti-CD63 (Abcam), anti-CD81 (Abcam), anti-SK1 (Abcam), anti-SP1 (Abcam), and anti-GAPDH (Santa Cruz Biotechnology). After four washes with PBS-Tween 20, horseradish peroxidase-conjugated secondary antibodies were added. The signals were detected with Pierce^®^ECL Western blotting substrate (Pierce, Rockford, IL, USA) and developed on X-ray films (Kodak, Rochester, NY, USA).

### Statistical analysis

Continuous variables are presented as mean ± SEM. Analysis of variance (ANOVA) and post hoc Bonferroni analysis was conducted for multiple comparisons by GraphPad Prism 5.0 (GraphPad Software, Inc., La Jolla, CA, USA). *p*-Values <0.05 are considered statistically significant.
